# Strongyloidiasis: the most neglected tropical disease in Ethiopia

**DOI:** 10.1186/s40249-021-00851-2

**Published:** 2021-05-07

**Authors:** Abebaw Tiruneh, Endalew Zemene, Zeleke Mekonnen

**Affiliations:** School of Medical Laboratory Sciences, Institute of Health, Jimma University, Jimma, Ethiopia

**Keywords:** Strongyloidiasis, Neglected tropical disease, Ethiopia

## Abstract

**Background:**

Strongyloidiasis is the most neglected of the neglected tropical diseases (NTDs). The aim of this commentary is to describe the possible reasons why strongyloidiasis is so overlooked in Ethiopia, and shed light on better ways of control and elimination of the disease.

**Main body:**

This commentary highlights three points why strongyloidiasis is the most neglected of the NTDs in Ethiopia. Firstly, lack of clear category within the NTDs resulted in omission of the disease from reports, intervention programs, and preventive chemotherapy guidelines. Secondly, magnitude of the disease is underestimated due to paucity of studies and low sensitivity of diagnostic methods coupled with asymptomatic nature of most of the infections. Finally, ivermectin (the drug of choice for treatment of strongyloidiasis) is not in use for control of the other soil-transmitted helminthiasis, nor is there ivermectin mass drug administration for control of strongyloidiasis. This might have created gap in control and elimination of the disease in Ethiopia and possibly elsewhere.

**Conclusion:**

Strongyloidiasis appears to be the most neglected of the NTDs mainly due to nature of the infection, low sensitivity of the routine diagnostic tools and it’s exclusion from strategic plans and intervention programs. Moreover, studies on strongyloidiasis should use sensitive diagnostic tools. Strongyloidiasis control and elimination programs should be based on reliable evidence of epidemiology of the disease in Ethiopia.

**Graphic Abstract:**

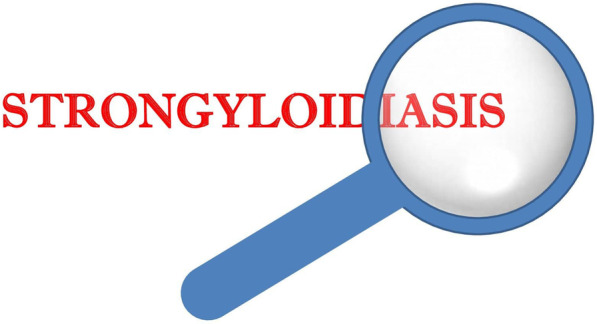

## Background

Strongyloidiasis is a parasitic disease caused by *Strongyloides stercoralis*. It is the most neglected soil-transmitted helminthiasis within the neglected tropical diseases (NTDs). The global burden of the disease is underestimated due to lack of precise data from endemic countries. However, a recent literature review estimated that about 613.9 million people were infected with *S. stercoralis* in 2017 [1]. More than 76% of the global burden of strongyloidiasis occurs in South-east Asia, Africa, and Western Pacific regions. The disease is ubiquitous though information is limited in some countries [2].

Ethiopia is among countries with high prevalence of *S. stercoralis* [2]. Most of the studies done in Ethiopia reported prevalence of *S. stercoralis* with other intestinal parasites utilizing diagnostic methods of low sensitivity to detect the parasite in stool samples [3]. This may result in under-estimation of strongyloidiasis in the country. Thus, the magnitude of the disease is likely far beyond what has been reported in the literatures. Moreover, burden of the disease is not known at national level; hence, making strongyloidiasis the most ignored of the NTDs in Ethiopia. This commentary describes possible reasons why strongyloidiasis is the most overlooked of the NTDs in Ethiopia and highlights the way forward for better control and elimination of the disease together with soil-transmitted helminthiasis and other NTDs. Strongyloidiasis is the most neglected tropical disease in Ethiopia, at least due to the following reasons:

### Lack of clear category of strongyloidiasis in the NTDs group

*S. stercoralis* is theoretically grouped under the soil-transmitted helminths (STHs) with *Ascaris lumbricoides*, *Trichuris trichiura* and hookworms (*Ancylostoma duodenale* and *Necator americanus*) in the list of NTDs. The 2030 targets for the STHs control program recognized existing gaps and underlined how to proceed with control and elimination of *Strongyloides*, including identification of endemic regions and ivermectin mass drug administration (MDA) [4]. The 2021–2030 road map for NTDs also give emphasis to strongyloidiasis and planned surveillance, ivermectin MDA, and establishment of easy diagnostic methods [5]. The current attention given to the disease is remarkable improvement and is a milestone for the future control and elimination programs like other STHs.

In contrast, previous reports, control programs, and preventive chemotherapy (PC) guidelines often do not consider strongyloidiasis as part of the soil-transmitted helminthiasis. The 2011–2020 strategic plan of elimination of STHs as public health problem in children focused only on *A. lumbricoides*, *T. trichiura* and hookworms [6]. The next fear of overlooking strongyloidiasis from the NTDs would mean missing this disease from plan of ‘Ending the neglect to attain Sustainable Development Goals: A road map for NTDs 2021–2030 [7]. The road map listed all NTDs except strongyloidiasis and justified specific and collective intervention programs. Therefore, global plans/programs targeting control and elimination of NTDs should adhere to ‘A road map for NTDs 2021–2030’ [5] to include strongyloidiasis in their future programs, reports, and plans as a disease of public health importance.

Ethiopian Ministry of Health identified nine priority NTDs comprising STHs, schistosomiasis, trachoma, lymphatic filariasis (LF), onchocerciasis, leishmaniasis, podoconiosis, dracunculiasis and scabies [8]. Although Ethiopia is among strongyloidiasis endemic countries [2], this disease is not included in the national STHs mapping and MDA programs. It was also omitted from the list of NTDs in steps to mobilize resources to achieve 2020 goals for the control and elimination of NTDs in Ethiopia, national reports, and implementation programs of STHs and/or NTDs [9, 10]. Strongyloidiasis got less attention in Africa compared to other endemic countries. Lessons could be learnt from the Americas, Cambodia and Oceania which included strongyloidiasis as one of STHs and conducting interventional studies, and implementing control and elimination programs [2, 11, 12]. Therefore, the Ministry of Health’s emphasis should be on developing national endemicity mapping and appropriate intervention for the control and elimination of the disease with other NTDs. Furthermore, the country needs to actively participate in ‘A road map for NTDs 2021–2030’ [5] in collaboration with World Health Organization (WHO) and other stakeholders in the future control and elimination programs.

### Low sensitivity of conventional STHs diagnostic methods

Strongyloidiasis is one of the opportunistic parasitic diseases, and majority of infected individuals are asymptomatic. This may result in decreased treatment seeking, hence, may enhance transmission of the disease. Moreover, the gold standard diagnostic tool for the detection of *S. stercoralis* in clinical samples is not yet available.

The main diagnostic stage for *A. lumbricoides*, *T. trichiura*, and hookworms in stool samples is the ova, while the diagnostic stage for *Strongyloides* is usually the larvae. Conventional diagnostic methods used in epidemiological studies of the other STHs, including Kato-Katz, McMaster, and Mini-FLOTAC, have low sensitivity for detecting *Strongyloides* larvae in stool samples. On the other hand, diagnostic tools such as modified formol-ether concentration, Baermann funnel, agar plate culture (APC), serology, and polymerase chain reaction (PCR) have relatively higher sensitivity, and where possible such specific techniques need to be employed for *Strongyloides* to increase the diagnostic sensitivity [13].

The WHO already recognized lack of standardized diagnostic methods and held a virtual meeting on ‘Diagnostic methods for the control of strongyloidiasis’ on September 29, 2020 [14]. During this meeting, the suitability of the currently available diagnostic methods (coprological, serological and PCR) to estimate prevalence of the disease at population level was assessed. Serological diagnostic methods particularly, recombinant *Strongyloides* antigen (NIE) enzyme-linked immunosorbent assay was proposed as the best diagnostic choice in combination with coprological tests, Baermann and APC.

In Ethiopia, direct stool smear microscopy is the diagnostic method for detecting *S. stercoralis* in health facilities and epidemiological studies. However, the diagnostic sensitivity of single direct smear microscopy is only about 30%. Formol-ether concentration technique is also used in epidemiological studies of STHs in Ethiopia. This method may increase the yield of *Strongyloides* larvae compared to direct smear microscopy but still has low sensitivity. Only few studies conducted in limited areas of Ethiopia used Harada-Mori technique, Baermann concentration, APC, water emergence technique, and PCR. Using these diagnostic methods, studies conducted in North-west Ethiopia, Southern Ethiopia, and Addis Ababa documented prevalence of *S. stercoralis* ranging from 12 to 20.7% [3]. These results indicate that the prevalence of the disease in Ethiopia is likely underestimated due to the low sensitivity of diagnostic methods utilized in the studies. Hence, it demands for uses of sensitive and specific diagnostic methods to accurately estimate the burden of *S. stercoralis* at national level.

### Distinct drug of choice for *S. stercoralis* and the STHs

School-based MDA with albendazole/mebendazole is underway for the control and elimination of STHs in Ethiopia. However, since the drug of choice for strongyloidiasis is ivermectin, the current MDA which did not include ivermectin in the national program of MDA for STH may not be effective against strongyloidiasis [4]. This might be partly attributed to the fact that ivermectin is only donated by Merck and Co., Inc., Kenilworth NJ USA, the Mectazin Donation Program (MSD) for the control and elimination program of onchocerciasis and LF [15]. Considering this limitation, WHO is currently supporting generic producers of which, the first prequalified ivermectin produced by Edenbridge Pharmaceuticals LLC–USA is on pipeline [16]. This might improve the accessibility of the drug at an affordable price in the market in the near future.

On the other hand, ivermectin has been used in MDA program for elimination of onchocerciasis and LF in Ethiopia. This might have significant collateral impact on reducing the burden of strongyloidiasis in co-endemic areas [10]. Nevertheless, transmission of *S. stercoralis* may continue in areas where onchocerciasis and/or LF are non-endemic. Therefore, identification of strongyloidiasis endemic areas and implementation of control and elimination program including ivermectin MDA for at-risk group needs to be considered. Moreover, it is also essential to evaluate the collateral impact of ivermectin MDA for the control and elimination of onchocerciasis and/or LF on epidemiology of strongyloidiasis.

## Conclusions

Clear category in the NTDs group and inclusion of strongyloidiasis in strategic plan, intervention programs, and reports are required for control and elimination of strongyloidiasis in endemic countries, including Ethiopia. National mapping of strongloidiasis using sensitive detection methods is essential.  Moreover, ivermectin MDA and other intervention programs are needed for the control and elimination of *S. stercoralis* with other NTDs in Ethiopia.

## Data Availability

Not applicable.

## Abbreviations

APC: Agar plate culture
LF: Lymphatic filariasis
MDA: Mass drug administration
NTDs: Neglected tropical diseases
PC: Preventive chemotherapy
PCR: Polymerase chain reaction
STHs: Soil-transmitted helminths
WHO: World Health Organization
